# A Source of Terrestrial Organic Carbon to Investigate the Browning of Aquatic Ecosystems

**DOI:** 10.1371/journal.pone.0075771

**Published:** 2013-10-04

**Authors:** Jay T. Lennon, Stephen K. Hamilton, Mario E. Muscarella, A. Stuart Grandy, Kyle Wickings, Stuart E. Jones

**Affiliations:** 1 Department of Biology, Indiana University, Bloomington, Indiana, United States of America; 2 W. K. Kellogg Biological Station and Department of Zoology, Michigan State University, Hickory Corners, Michigan, United States of America; 3 Department of Natural Resources and the Environment, University of New Hampshire, Durham, New Hampshire, United States of America; 4 Department of Entomology, Cornell University, Geneva, New York, United States of America; 5 Department of Biological Sciences, University of Notre Dame, South Bend, Indiana, United States of America; University of California, Merced, United States of America

## Abstract

There is growing evidence that terrestrial ecosystems are exporting more dissolved organic carbon (DOC) to aquatic ecosystems than they did just a few decades ago. This “browning” phenomenon will alter the chemistry, physics, and biology of inland water bodies in complex and difficult-to-predict ways. Experiments provide an opportunity to elucidate how browning will affect the stability and functioning of aquatic ecosystems. However, it is challenging to obtain sources of DOC that can be used for manipulations at ecologically relevant scales. In this study, we evaluated a commercially available source of humic substances (“Super Hume”) as an analog for natural sources of terrestrial DOC. Based on chemical characterizations, comparative surveys, and whole-ecosystem manipulations, we found that the physical and chemical properties of Super Hume are similar to those of natural DOC in aquatic and terrestrial ecosystems. For example, Super Hume attenuated solar radiation in ways that will not only influence the physiology of aquatic taxa but also the metabolism of entire ecosystems. Based on its chemical properties (high lignin content, high quinone content, and low C:N and C:P ratios), Super Hume is a fairly recalcitrant, low-quality resource for aquatic consumers. Nevertheless, we demonstrate that Super Hume can subsidize aquatic food webs through 1) the uptake of dissolved organic constituents by microorganisms, and 2) the consumption of particulate fractions by larger organisms (i.e., *Daphnia*). After discussing some of the caveats of Super Hume, we conclude that commercial sources of humic substances can be used to help address pressing ecological questions concerning the increased export of terrestrial DOC to aquatic ecosystems.

## Introduction

Aquatic ecosystems are connected to the surrounding landscape through inputs of material and energy from terrestrial ecosystems. It is estimated that inland water bodies receive nearly three petagrams of terrestrial carbon on an annual basis [1], [2], [3]. The majority of this organic matter, comprised of humic substances derived from vascular plants, is delivered to streams, lakes, and estuaries in the form of dissolved organic carbon (DOC). Terrestrial DOC influences the physical, chemical, and biological features of recipient aquatic ecosystems in many ways. For example, the chromophoric properties of terrestrial DOC can affect the physiology and behavior of aquatic organisms by reducing UV stress [4], and at the same time, reduce rates of primary productivity via the attenuation of photosynthetically active radiation [5]. In addition, terrestrial DOC forms complexes with trace elements and nutrients, which influences the turnover and bioavailability of these resources for aquatic biota [6], [7]. Last, terrestrial DOC is made up of allochthonous compounds that can subsidize aquatic food webs [8] and regulate net ecosystem production [9].

Growing evidence suggests that DOC concentrations in aquatic ecosystems are rising in certain parts of the world [10], [11]. It has been hypothesized that this “browning” trend may be associated with drivers of global change, including altered land-use [12], precipitation patterns [13], temperatures [14], and atmospheric deposition [15]. Regardless of the controlling factors, there is considerable uncertainty about how aquatic ecosystems will respond to increasing inputs of terrestrial DOC [16]. Insight about the effects of browning can be gained from comparative studies [17], but these space-for-time substitutions are correlative and may not capture the full range of interactions that aquatic ecosystems will experience in the future.

Experimental approaches provide an opportunity to address uncertainty about global change scenarios, including the effects of browning. However, it is challenging for aquatic scientists to obtain enough terrestrial DOC to conduct manipulative studies at ecologically relevant scales. For small-scale experiments (∼1L), it is possible to extract milligram to gram quantities of natural humic substances to address questions related to microbial activity over short periods of time [18]. But larger experimental units (and more DOC) are required if one is interested in elucidating the interactive effects that terrestrial DOC has on food webs, physical processes, and ecosystem functioning. Terrestrial DOC has been manipulated in aquatic mesocosms (∼10–1,000 L) through the addition of leachates from soils [19], deciduous leaves [20], or peat moss [21]. The downside of this approach, however, is that leaching sometimes involves artificial processing of the organic material [22], [23]. In addition, the chemical composition of freshly leached DOC may be distinct from the older humic substances that typically dominate DOC in aquatic ecosystems [24]. Browning can also be simulated by adding water from humic-rich systems to mesocosms containing low DOC water from another system [25], [26], but this mixing approach may be confounded by the transfer of organisms and unintended effects on mesocosm water chemistry. Last, terrestrial DOC can be manipulated at the ecosystem scale. For example, concentrations of terrestrial DOC have been indirectly elevated by altering hydrological flows through the installation of curtains in relatively small lakes [27], [28]. And in one rather unique case, 23,000 L of humic water originating from the Suwanee River (Georgia, USA) was transported nearly 3,000 km in a tanker truck before being released into two high elevation rivers (Colorado, USA) [29]. Unfortunately, these types of large-scale manipulation are not always practical or easy to control, nor are they amenable to replication [30].

As an alternative to the experimental approaches described above, DOC manipulations could potentially be performed at a range of scales using concentrated solutions of humic substances, which can readily be obtained in large quantities from commercial suppliers. Humic substances are commonly used as an amendment in agricultural ecosystems to increase soil water-holding capacity, reduce erosion, improve pH buffering capacity, and stimulate the activity of beneficial microorganisms [31]. Many of the commercially available sources of humic substances are derived from Leonardite, a highly oxidized mineral-like substance that is extracted from geologic deposits in North America and other parts of the world [32]. Previous studies have investigated the chemical and physical properties of Leonardite-derived materials to understand how humic substances affect the behavior of metals and the solubility of organic pollutants [33], [34], [35]. It is yet to be determined whether or not commercially available humic substances might serve as an adequate analog for addressing ecological questions about the importance of terrestrial DOC in aquatic ecosystems. Therefore, in this study, we used a combination of analytical procedures, laboratory assays, and comparative surveys to describe the chemical and physical characteristics of a commercially available source of humic substances. In the process, we identify a putative set of heterotrophic bacteria that are capable of growing on humic substances and report results from a series of whole-pond manipulations that explore the relationship between DOC loading and microbial metabolism. Last, we discuss the opportunities and caveats of using commercially available sources of humic substances for addressing ecological questions about terrestrial DOC in aquatic ecosystems.

## Materials and Methods

### Background

We compared the properties of a commercially available source of humic substances to DOC in natural ecosystems using various approaches, including whole-ecosystem manipulations. Specifically, we altered the supply rate of humic substances to 11 ponds at the Kellogg Biological Station (KBS) Experimental Pond Facility at Michigan State University. Each of the vinyl-lined earthen ponds measures 30 m in diameter, has a maximum depth of 2 m, and an operating volume of approximately 10^6^ L. Based on concentrations of total phosphorus, the ponds are considered mesotrophic (0.48–0.65 µmol P L^−1^) and contain plankton assemblages that are typical of lentic habitats in the region [36]. Prior to initiating our experiment, we filled each of the ponds with water from a groundwater well and allowed approximately one month for acclimation. We then established a gradient of terrestrial carbon supply (0 to 177 mmol C m^−2^ d^−1^) by adding different quantities of a commercially available source of humic substances to each pond. The humic substances used in our study came from a product called “Super Hume”, which is marketed by CropMaster, United Agricultural Services of America, Inc. (Lake Panasoffkee, Florida, USA). Super Hume is extracted from Leonardite shale, a naturally occurring oxidized form of lignite coal. The extraction involves treatment with potassium hydroxide, which yields a basic (pH = 11.5) and concentrated (15% fulvic acid and 4% humic acid, dry mass) solution of humic substances. Super Hume was shipped to the Experimental Pond Facility in 1,000 L carboys and added to each pond on a weekly basis using a gas-powered pump. We sampled water from the center of each non-aerated pond in a jon boat using a 1-m depth-integrated tube sampler three times per week for approximately 14 weeks from early June to mid September 2009. We monitored various limnological variables during our sampling. Some of these methods and results are discussed below, while other findings are the focus of another manuscript [37].

### Physical Characteristics: Size Distribution

The size distribution of humic substances is important for determining the physical, chemical, and biological behavior of organic matter in aquatic ecosystems. To assess the size distribution of Super Hume at a relatively coarse resolution, we first quantified the total organic carbon concentration of Super Hume solutions (n = 3) diluted in Milli-Q filtered water (target concentration = 2 mmol L^−1^) via oxidation and subsequent nondispersive infrared (NDIR) detection using a Shimadzu TOC-V carbon analyzer. We then measured the amount of organic carbon in different size classes based on the concentration of organic carbon that passed through Whatman (Kent, UK) glass microfiber filters (GF/D = 2.7 µm and GF/F = 0.7 µm) and Supor (Pall Corporation, Port Washington, NY, USA) polyethersulfone (PES) membrane filters (0.2 µm). This filtration process allowed us to quantify the relative amounts of Super Hume in four size classes: <0.2 µm; 0.2–0.7 µm; 0.7–2.7 µm; >2.7 µm.

### Physical Characteristics: Flocculation

At the beginning of our field experiment, we observed that DOC concentrations in the experimental ponds receiving Super Hume declined at a faster rate than we had anticipated. This pattern suggested that Super Hume was undergoing flocculation, a process whereby DOC aggregates into particulate organic carbon (POC), which is subject to sinking. To test our hypothesis, we estimated flocculation rates for Super Hume under laboratory conditions. We added different amounts of Super Hume to nine 1-L polycarbonate bottles containing unfiltered water from a KBS reference pond. Flocculation was estimated as the loss of DOC (<0.7 µm) in non-agitated bottles that were incubated at room temperature for four days. We did not account for biological production or consumption of DOC during the incubations. The flocculation rate constant was quantified by regressing the DOC flocculation rate (µmol L^−1^ d^−1^) against the initial DOC concentration (µmol L^−1^). To assess the potential for background water chemistry to affect sedimentation, we also estimated flocculation with water collected from six lakes (North Gate Pond, Morris Lake, Roach Lake, Kickapoo Lake, Peter Lake, Long Lake) at the University of Notre Dame Environmental Research Center (UNDERC) in northern Wisconsin. For these assays, flocculation rates were only measured at a single Super Hume concentration (667 µmol DOC L^−1^) as opposed to the full gradient (0–3,333 µmol DOC L^−1^) for water from the KBS reference pond.

### Physical Characteristics: Optical Properties

We assessed the light-absorbing properties of Super Hume by comparing color and Secchi depth along DOC gradients in the KBS experimental ponds and natural lakes. Color was quantified on surface water samples spectrophotometrically using a 10 cm quartz cuvette and expressed as a_440_, where a_440_ = 2.303 (absorbance at 440 nm/0.1 m) [38] while Secchi depth was measured following standard procedures [39]. For natural systems, data for color and Secchi depth were obtained from lakes in New England and upstate New York [17], Wisconsin [40], and Michigan (Lennon unpublished data). For the experimental ponds, we used time-averaged estimates for color (n = 36) and Secchi depth (n = 12). We then compared the color-DOC and Secchi-DOC relationships using indicator variables multiple regression [41] where DOC was treated as a continuous predictor variable and ecosystem type (experimental pond vs. natural lake) was treated as a categorical predictor variable. We log_10_-transformed our data when necessary to help meet the assumptions of normality and equal variance. Differences in the slopes or intercepts between ecosystem types would suggest that Super Hume differentially affected the optical properties of water when compared to natural sources of DOC.

### Chemical Characteristics: Elemental Ratios

The elemental composition of terrestrial organic matter is an important feature that can influence resource quality for aquatic consumers. Therefore, first, we measured total nitrogen (TN) and total phosphorus (TP) after persulfate digestion using spectrophotometric analysis [42]. Second, we analyzed the concentrations of major cations and anions on a diluted Super Hume sample (800 µmol DOC L^−1^) using a Dionex membrane-suppression ion chromatograph. Last, we estimated total alkalinity by acid titration, as well as by charge balance of the major cations and anions. We then compared the elemental chemistry of Super Hume to observations from a database of streams and rivers (n = 78), groundwater samples (n = 58), lakes (n = 111), wetlands (n = 134), and soil-water samples (n = 142) that were collected in southwestern Michigan between 1996–2009 using methods described elsewhere [43], [44], [45]. In order to compare the chemistry of Super Hume to environmental samples, we standardized the molar concentration (or equivalents, in the case of alkalinity) by the molar DOC concentration of each sample.

### Chemical Characteristics: Organic Properties

We characterized the organic chemical properties of Super Hume using two methods. First, we compared Super Hume to aquatic and terrestrial reference materials using pyrolysis-gas chromatography/mass spectrometry (py-GC/MS). The seven reference materials consisted of four soil samples, an algal sample, a DOC sample from a eutrophic lake (Wintergreen Lake, MI), and a DOC sample from a dystrophic lake (Brandywine Lake, MI). The soil reference samples were fine-loamy, mixed, mesic typic Hapludalfs obtained from surface soils of agricultural sites located near the KBS Experimental Pond Facility. The algal sample was prepared from a laboratory culture of *Ankistrodesmus* sp., which was dried at 60°C before pyrolysis, while carbon from the lake DOC samples was obtained by collecting organic matter after evaporating 1 L of 0.7 µm-filtered water samples. A detailed description of the py-GC/MS methods can be found elsewhere [46]. Briefly, after sample pyrolysis at 600°C, compounds were separated and identified using gas chromatography and ion trap mass spectrometry. Peaks were identified using the National Institute of Standards and Technology (NIST) compound library and were subsequently binned into six primary chemical classes (lignin, lipids, phenols, nitrogen-bearing compounds, polysaccharides, and compounds of unknown origin). Data are reported as relative abundances and represent proportions of the total ion signal characterized during analysis. We visualized the multivariate data for the different samples using Principal Coordinates Analysis (PCoA) with a Bray-Curtis distance matrix. We used the *envfit* function in the vegan package of R [47] to project vectors onto our ordination to visualize correlations between chemical attributes and our reference samples.

Second, we characterized Super Hume using fluorescence spectroscopy and parallel factor analysis (PARAFAC). Using a Perkin Elmer LS50B fluorometer, we generated excitation-emission matrix spectra (EEM) from a 3D fluorescence scan (excitation: 240–450 every 10 nm; emission 350–550 every 2 nm) on a diluted Super Hume sample (883 µmol C L^−1^). We corrected the EEM by implementing manufacturer-supplied correction files and by subtracting the resulting values from Milli-Q water blanks. Prior to analysis, we normalized the data to the area under the water Raman peak (excitation 350 nm) [48]. We then fit the Super Hume fluorescence data to a preexisting and validated PARAFAC model, which statistically decomposed the EEM spectra of the sample into loading components related to the organic matter constituents. This model yields 13 loading components based on 379 samples from diverse aquatic habitats [49]. We used the model fit, residuals, and loadings of the 13 model components to characterize the organic matter properties of Super Hume relative to other surface waters.

### Chemical Characteristics: Isotopic Composition

Because they can be used to help track the fate of carbon in food webs and ecosystems, we quantified the stable and radioactive carbon isotope ratios of Super Hume. Dried Super Hume was analyzed for δ^13^C at the University of California Davis Stable Isotope Facility with a PDZ Europa trace gas analyzer and a continuous-flow Europa 20/20 isotope ratio mass spectrometer (IRMS). The Δ^14^C of Super Hume was estimated from graphite targets at the Center for Accelerator Mass Spectrometry at Lawrence Livermore National Laboratory after subtracting background measurements of ^14^C-free coal [50].

### Biological Responses: Zooplankton Life History

We observed what appeared to be humic substances in the guts of zooplankton from a Super Hume-enriched pond. To evaluate the potential effects that Super Hume might have on zooplankton fitness, we conducted a life table experiment using a clone of *Daphnia pulex x pulicaria* isolated from a KBS pond that had received Super Hume over the course of the growing season (177 mmol C m^−2^ d^−1^). Prior to initiating the life table experiment, we propagated the *Daphnia* clone for multiple generations at room temperature in COMBO medium [51] and fed the animals with a culture of green algae (*Ankistrodesmus* sp.).

To initiate the life table experiment, we randomly assigned 15 neonates to a control treatment without Super Hume (−SH) and 15 neonates to a Super Hume treatment (+SH) containing 1,666 µmol DOC L^−1^. We then followed the methods for life table analysis described in detail elsewhere [52]. Briefly, individual *Daphnia* were grown in 70 mL tissue-culture flasks containing COMBO medium and 20,000 cells mL^−1^ of *Ankistrodesmu*s in an environmental chamber (25°C, 16∶8 light-dark cycle) for 21 days. Each day, we recorded whether an individual was alive or dead (survivorship) and the number of neonates that were produced (reproduction). At this time, we also transferred the focal daphnid to a new flask containing fresh medium, food, and Super Hume (depending on the treatment).

We used Euler’s method to calculate the intrinsic rate of increase, *r* (d ^−1^): 1 = Σ e−*^r^* ^x^ *l*(x) *b*(x), where *b*(x) is number of offspring produced per individual on day x, and *l*(x) is the proportion of individuals in a treatment surviving to the next day. We estimated the standard error for the intrinsic rate of increase using jackknifing procedures [53]. In addition, we calculated the net reproductive rate (*R*_o_) as the sum of offspring produced for individuals and generation time as Σx *l*(x) *b*(x)/Σ *l*(x) *b*(x).

Last, we estimated the ingestion rates and assimilation rates of *Daphnia* in the −SH and +SH treatments by feeding animals suspensions of *Ankistrodesmus* (50,000 cells mL^−1^) that had been labeled with H^14^CO_3_^−^ [54]. We measured ingestion rates based on the counts per minute (CPM) of individual *Daphnia* after two hours of feeding. Assimilation rates were estimated by quantifying the CPM of labeled *Daphnia* after they had incubated in COMBO medium without *Ankistrodesmus* for two hours. We then estimated assimilation efficiency as the ratio of assimilation rates to ingestion rates. We used a Kaplan-Meier test to determine the effects of Super Hume on *Daphnia* survivorship; for all other life history traits, we performed statistical comparisons using Student’s t-tests.

### Biological Responses: Humic Oxidizing Bacteria

We isolated bacteria from the experimental pond that received the highest supply rate (177 mmol C m^−2^ d^−1^) of Super Hume. Water was collected from the surface (0.5 m) of the pond in early August 2009 and immediately transported back to the lab where dilutions of the samples were spread onto washed agar plates (1.5%) containing a modified version of WC medium [55] (we substituted NH_4_Cl for KNO_3_ as the nitrogen source and did not include Na_2_SiO_3_ or H_2_O_3_Se). Super Hume was added to the plates at a final concentration of 3,333 µmol DOC L^−1^. We then watched for colony formation while incubating the plates in an aerobic chamber at 25°C in the dark for up to three weeks. To purify isolates, we picked single colonies and restruck them onto Super Hume plates before cryopreservation in 20% glycerol at −80°C. We identified the bacterial isolates by direct sequencing of the 16S rRNA gene. Briefly, DNA was extracted using the UltraClean® Microbial DNA Isolation Kit (Mo-Bio, Carlsbad, CA). We then used 5 ng of this DNA as a template in a polymerase chain reaction (PCR) using 8F and 1492R primers with thermal cycler conditions outlined elsewhere [56]. The resulting PCR products were sequenced at the Research Technology Support Facility (RTSF) at Michigan State University (East Lansing, Michigan, USA). We used the classifier tool in the Ribosomal Database Project [57] to identify each of the bacterial isolates. For visual purposes, we aligned our sequences in mothur [58] version 1.25.1 against the Silva reference database and generated a phylogenetic tree using the neighbor-joining algorithm in ClustalX (www.clustal.org). We deposited the 16S rRNA gene sequences in GenBank with the accession numbers JX312319– JX312328.

### Biological Responses: Microbial Metabolism

We examined the relationships between microbial metabolism and DOC supply in the experimental ponds that were enriched with Super Hume. We estimated bacterial productivity (BP) as the uptake and incorporation of ^3^H-leucine (50 nmol L^−1^ final concentration) into bacterial protein [59], bacterial respiration (BR) as the rate of dissolved oxygen depletion in GF/D-filtered (2.7 µm) samples using a Presens SensorDish Reader [60] and bacterial growth efficiency (BGE) as BP/(BP+BR). Microbial metabolism and DOC concentration were measured in each of the 11 ponds three times per week for 12 weeks. We evaluated the relationship between microbial metabolism and DOC using simple linear regression on time-averaged values.

## Results

### Physical Characteristics: Size Distribution

Super Hume contained a mixture of dissolved and fine particulate organic matter. Unfiltered Super Hume samples had a total organic carbon concentration of approximately 3.3 moles C L^−1^. The vast majority (80%) of this material could operationally be defined as DOC, with 40±6.0% in the <0.2 µm size class and 20±10.0% in the 0.2–0.7 µm size class. The remaining fraction of Super Hume (20%) could be operationally defined as particulate organic carbon (POC), with 11±1.0% in the 0.7–2.7 µm size class and 9±7.0% in the >2.7 µm size class. All values represent mean ± SEM.

### Physical Characteristics: Flocculation

**Figure 1:**
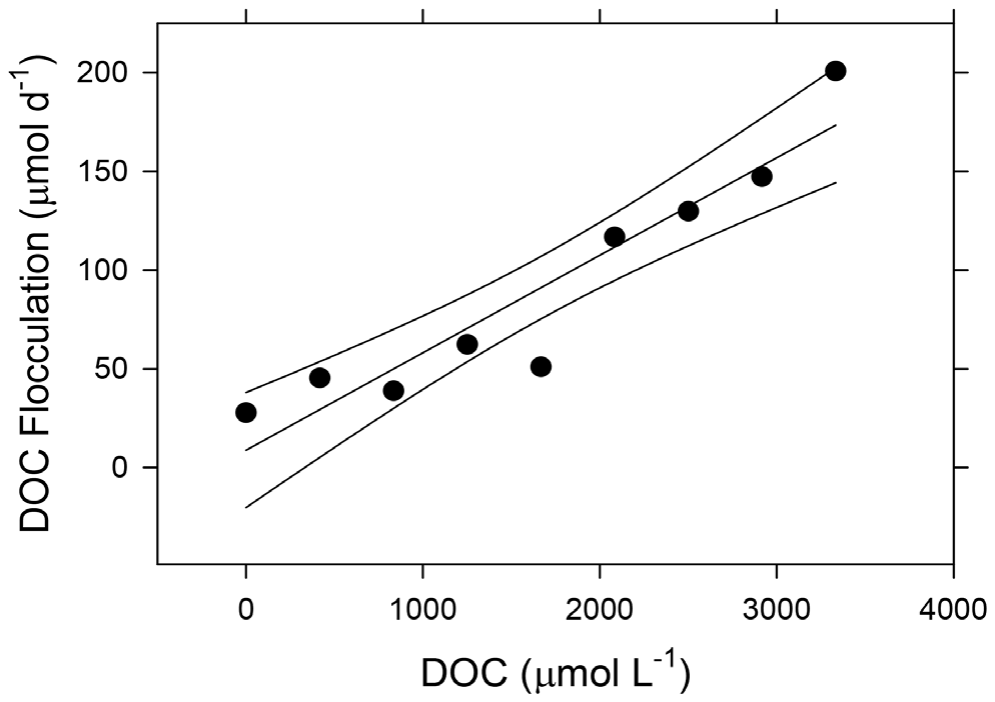
Flocculation rates of Super Hume. We estimated flocculation rates in laboratory assays as the loss of dissolved organic carbon (DOC) at different Super Hume concentrations added to water from a reference pond. Regression lines represent predicted values and 95% confidence intervals.

Our laboratory assays supported the hypothesis that Super Hume was undergoing flocculation in the experimental ponds. We observed that Super Hume was sedimenting to the bottom of the bottles during our incubations. This was confirmed by a significant loss of DOC over time from incubated pond water samples, which increased with Super Hume concentration (r^2^ = 0.89, F_1,10_ = 55.1, *P*<0.0001, Fig. 1):
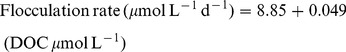
(1)


Our results indicate that 4.7–12.2% of the Super Hume standing stock was lost each day to sedimentation. However, the flocculation rates measured in northern Wisconsin lakes (4.5±0.92 µmol DOC L^−1^ d^−1^) at a given Super Hume concentration (667 µmol DOC L^−1^) were nine-fold lower than what was observed in the water from the experimental ponds (one sample t-test, t_5_ = −16.3, *P*<0.0001).

### Physical Characteristics: Optical Properties

In general, Super Hume and natural DOC had similar effects on the optical properties of aquatic ecosystems. The multiple regression model for color was highly significant (R^2^ = 0.84, F_3,110_ = 188, *P*<0.0001), but the slope and intercepts were affected by ecosystem type, which resulted in the following equations:
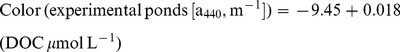
(2)


and
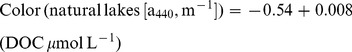
(3)


**Figure 2:**
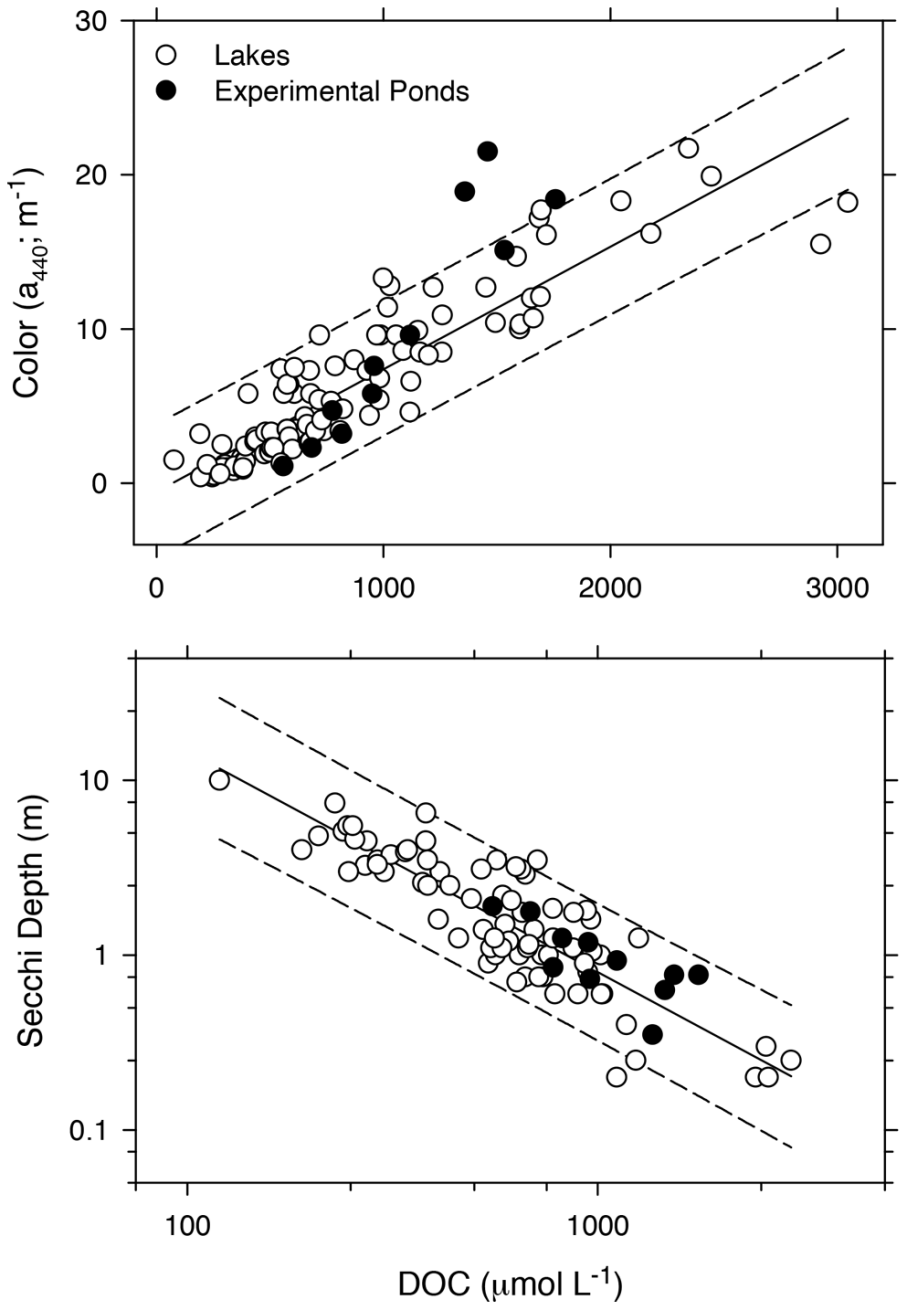
Super Hume effects on the aquatic light-environment. Comparison of color (A) and Secchi depth (B) as a function of DOC in natural lakes and experimental ponds. Variation in DOC concentration in the experimental ponds was achieved by manipulating the Super Hume supply rate. Regression lines represent predicted values and 95% prediction intervals.

Despite the differences in the regression parameters, most of the color observations from the experimental ponds fell within the 95% prediction intervals associated with the regression model for natural lakes in equation 3 (Fig. 2a).

Super Hume and natural DOC also had similar effects on Secchi depth, with no differences between natural lakes and experimental ponds (R^2^ = 0.76, F_3,84_ = 88.2, *P*<0.0001). Neither the slope nor the intercept was affected by ecosystem type, so a single model was used to fit the two data sets (Fig. 2b).
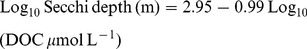
(4)


### Chemical Characteristics: Elemental Ratios

**Figure 3:**
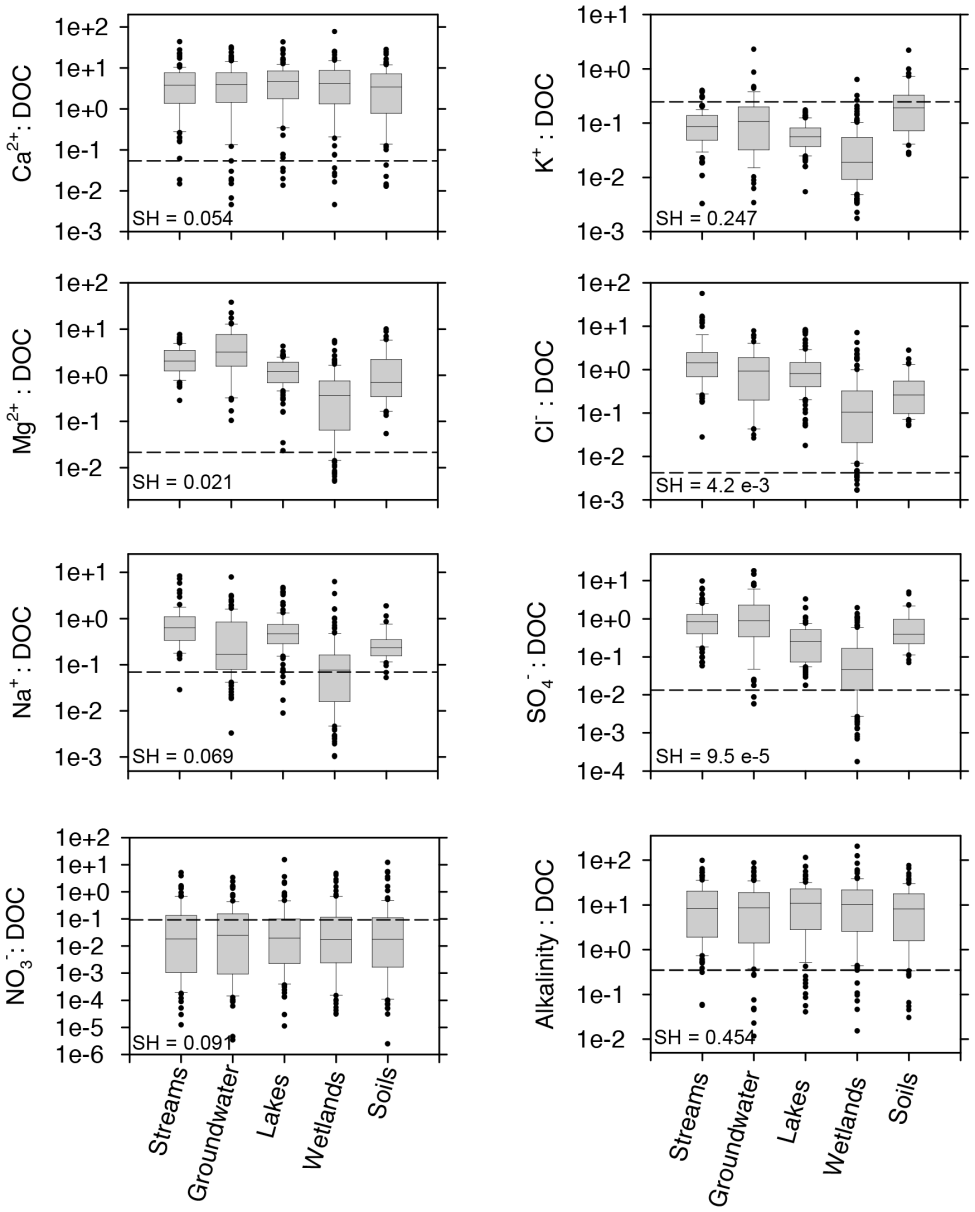
Super Hume stoichiometry. Molar ratios of major cation and anions with respect to DOC. We express alkalinity as equivalents per mole DOC. Observations for natural ecosystems (stream, groundwater, lakes, wetlands, and soil water) come from a survey of sites distributed throughout southwestern Michigan. Super Hume values are represented by the horizontal dashed line.

Super Hume is carbon-rich material with low nitrogen (N) and phosphorus (P) content (C:N = 440, C:P = 10,128 by moles). In addition, the N:P ratio of Super Hume was relatively high (276). With the exception of potassium (K), our results suggest that Super Hume amendments would only marginally affect the concentrations of major cations and anions found in streams, groundwater, lakes, wetlands, and soils in southwestern Michigan (Fig. 3). The K:DOC ratio of Super Hume was relatively high (0.247) compared to the median K:DOC ratios observed in wetlands (0.019), lakes (0.057), streams (0.087), groundwater (0.108), and soils (0.190). The alkalinity of Super Hume was 0.35±0.061 meq L^−1^ (mean ± SEM), which is relatively low compared to the alkalinity of natural systems (Fig. 3).

### Chemical Characteristics: Organic Properties

**Figure 4:**
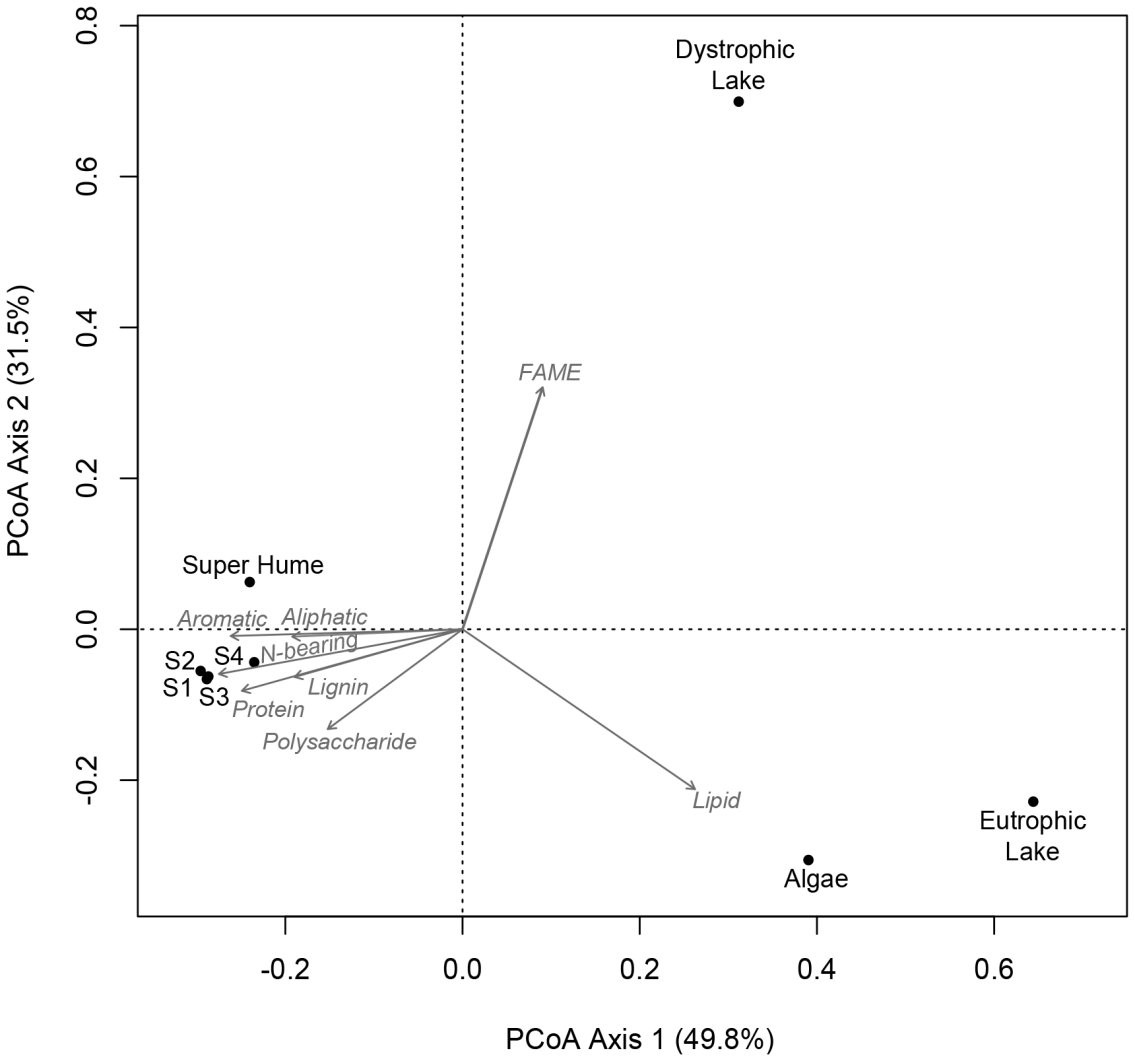
Multivariate ordination of Super Hume chemistry. We performed a Principal Coordinates Analysis (PCoA) based on the chemical characteristics of Super Hume and other reference organic materials as determined by pyrolysis-gas chromatography/mass spectrometry (py-GC/MS). Reference samples are represented by symbols, while vectors reflect correlations between chemical attributes and the samples.

The py-GC/MS data suggest that the chemical composition of Super Hume is similar to that of soil organic matter (Fig. 4). The first two axes of the principal coordinates analysis (PCoA) explained >80% of the variation in the py-GC/MS data. The first axis clearly separated soil samples from aquatic samples. Along this axis, Super Hume grouped closely with the soil samples, which had high relative abundances of lignin, protein, and polysaccharides. In contrast, algae and DOC from Wintergreen Lake (eutrophic) had a high lipid content, while the DOC from Brandywine Lake (dystrophic) was enriched with fatty acid methyl esters. The second axis indicated that the organic chemistries of DOC from the eutrophic and dystrophic lakes were distinct, while Super Hume was intermediate to these aquatic endmember samples.

**Figure 5:**
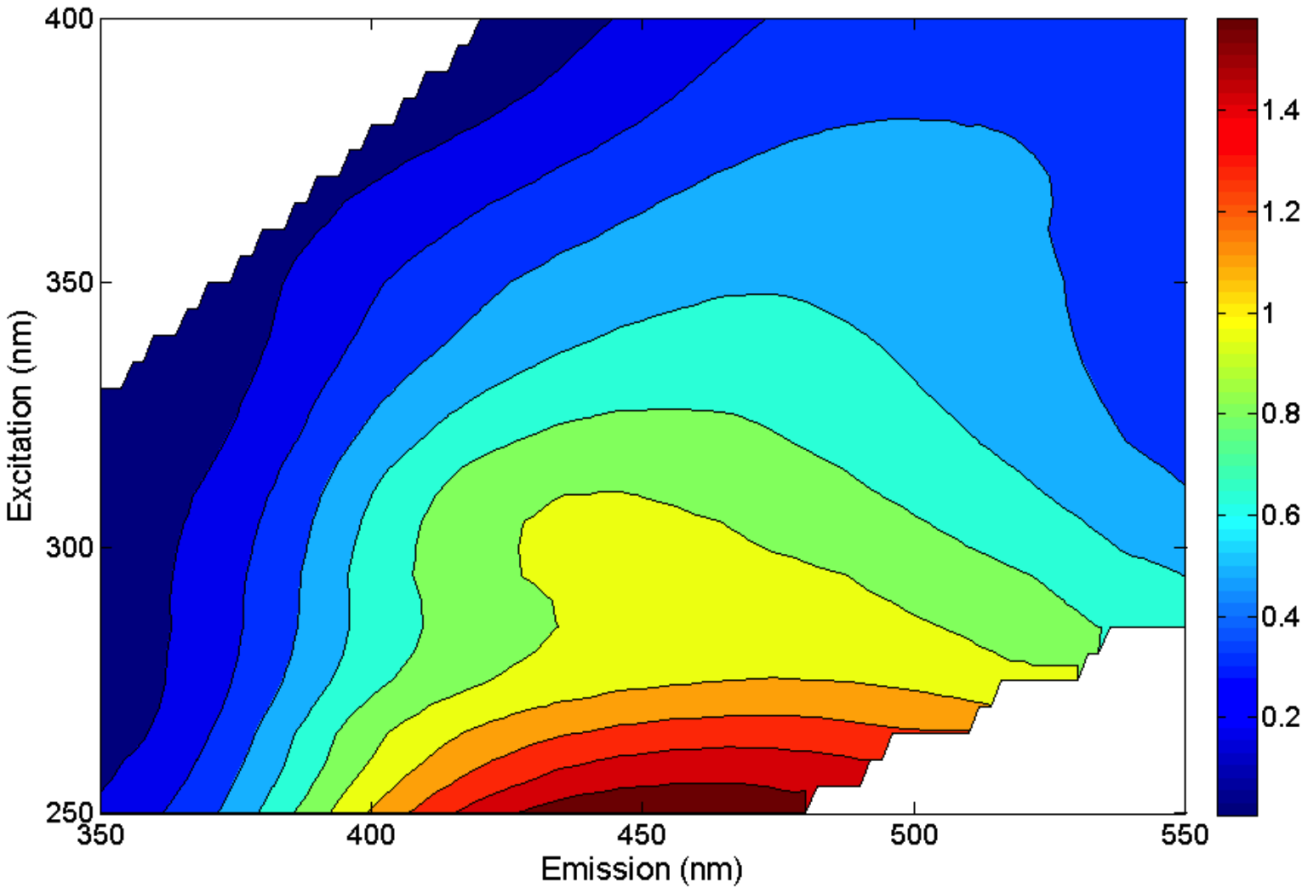
Heat map of Super Hume chemistry. The heat map represents the excitation-emission matrix spectra (EEMs) from a 3D fluorescence scan of Super Hume that was used in parallel factor analysis (PARAFAC).

Most of the variation (99%) in the fluorescent properties of the Super Hume EEM could be explained using the PARAFAC model (Fig. 5) [49]. The residual EEM values (differences between the raw EEM and model fit EEM) were low (<0.2%), indicating that there were no anomalous fluorophores in Super Hume. Oxidized quinone-like compounds (components 11, 2, 12) comprised 27% of the sample (7, 16, 4% respectively), while reduced quinone-like compounds (components 7, 9, 5, 4) comprised 49% of the sample. Component 4, which was a major component of the Super Hume sample (35%), is prevalent in EEMs of isolated humic substances derived from soils [61]. Only three of the 13 model components were not detected in Super Hume. Two of these (components 8 and 13), are indicative of amino acid-like molecules, while the other component (component 3) has been characterized as “unknown” and only recovered from Antarctic samples.

### Chemical Characteristics: Isotopic Composition

The δ^13^C of Super Hume was −23.6±0.48‰ (mean ± SEM, n = 4). The Δ^14^C was −984.8‰, which confirms that the carbon in Super Hume is ancient, as expected based on its origin from lignite.

### Biological Responses: Zooplankton Life History

**Figure 6:**
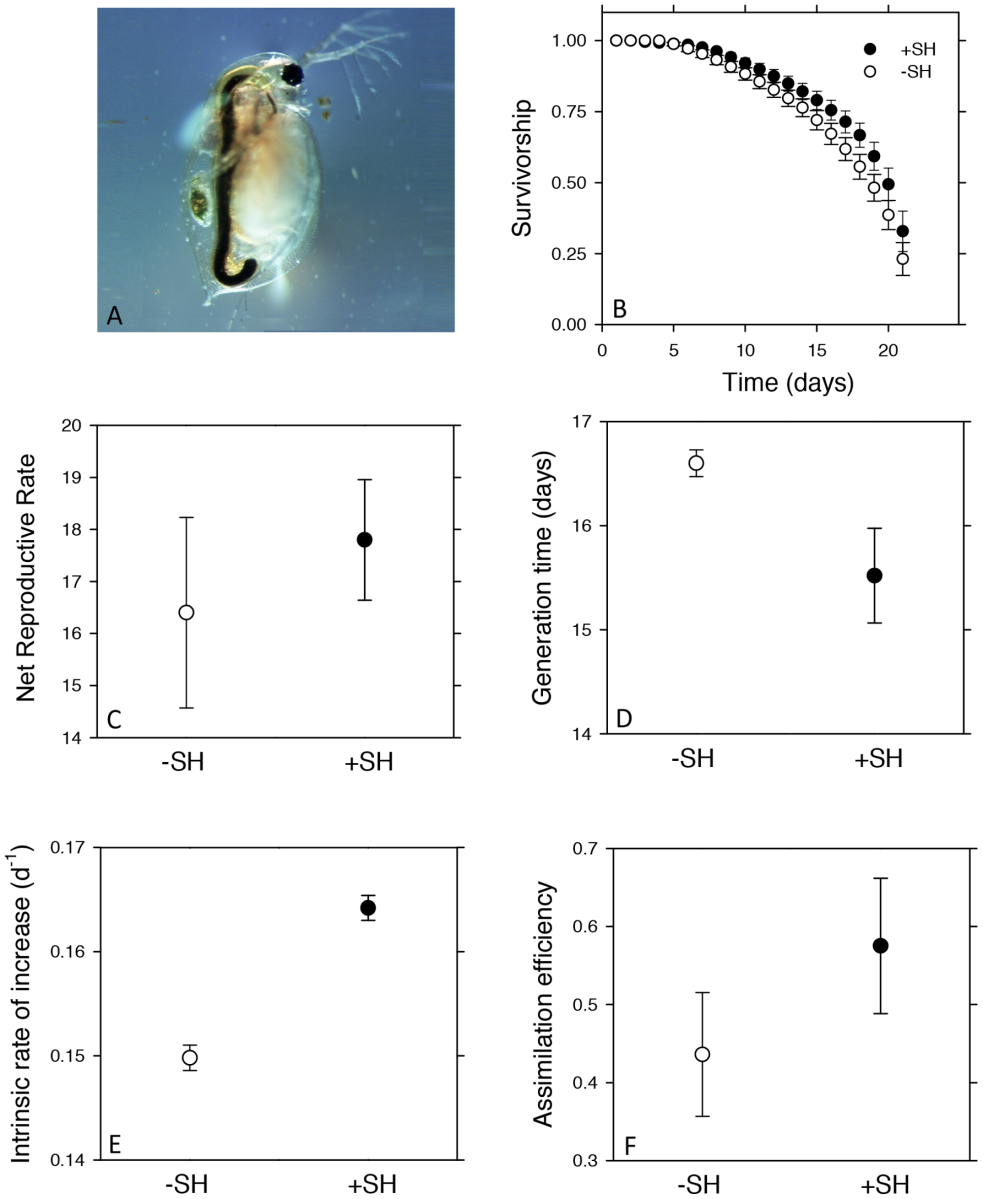
Effects of Super Hume on *Daphnia.* A) We observed the accumulation of humic substances in gravid individuals of *Daphnia pulex x pulicaria* collected from a pond that had been experimentally enriched with Super Hume. Subsequently, we measured the effects of Super Hume (1666 µmol L^−1^) on the life history (B–D), algal ingestion rates (E), and algal assimilation efficiency (F) where *D. pulex x pulicaria* was fed algae in the presence (+SH) or absence (−SH) of Super Hume. Values represent means ± SEM.

We did not find evidence from our life table experiment that *Daphnia pulex x pulicaria* was negatively affected by Super Hume, even at fairly high concentrations. Rather, some of our results suggest that Super Hume could potentially increase the fitness of *D. pulex x pulicaria*. For example, generation times were marginally reduced in the presence (15.5±0.455) vs. absence (16.6±0.129) of Super Hume (t_9.31_ = 2.18, *P* = 0.056, Fig. 6D), which contributed in part to an overall higher intrinsic rate of increase (−SH = 0.150±0.0012, +SH = 0.164±0.0012; t_28_ = −8.20, *P*<0.0001, Fig. 6E). These effects of Super Hume on population growth could not be attributed to differences in *D. pulex x pulicaria* ingestion rates (t_5.51_ = −0.252, *P* = 0.810) or assimilation efficiencies of algal carbon (t_5.95_ = −1.19, *P* = 0.281, Fig. 6F). Super Hume had no effect on survivorship (Chi-square = 1.30, *P* = 0.246, Fig. 6B) or net reproductive rate (t_13.5_ = −0.61, *P* = 0.550, Fig. 6C).

### Biological Responses: Humic Oxidizing Bacteria

**Figure 7:**
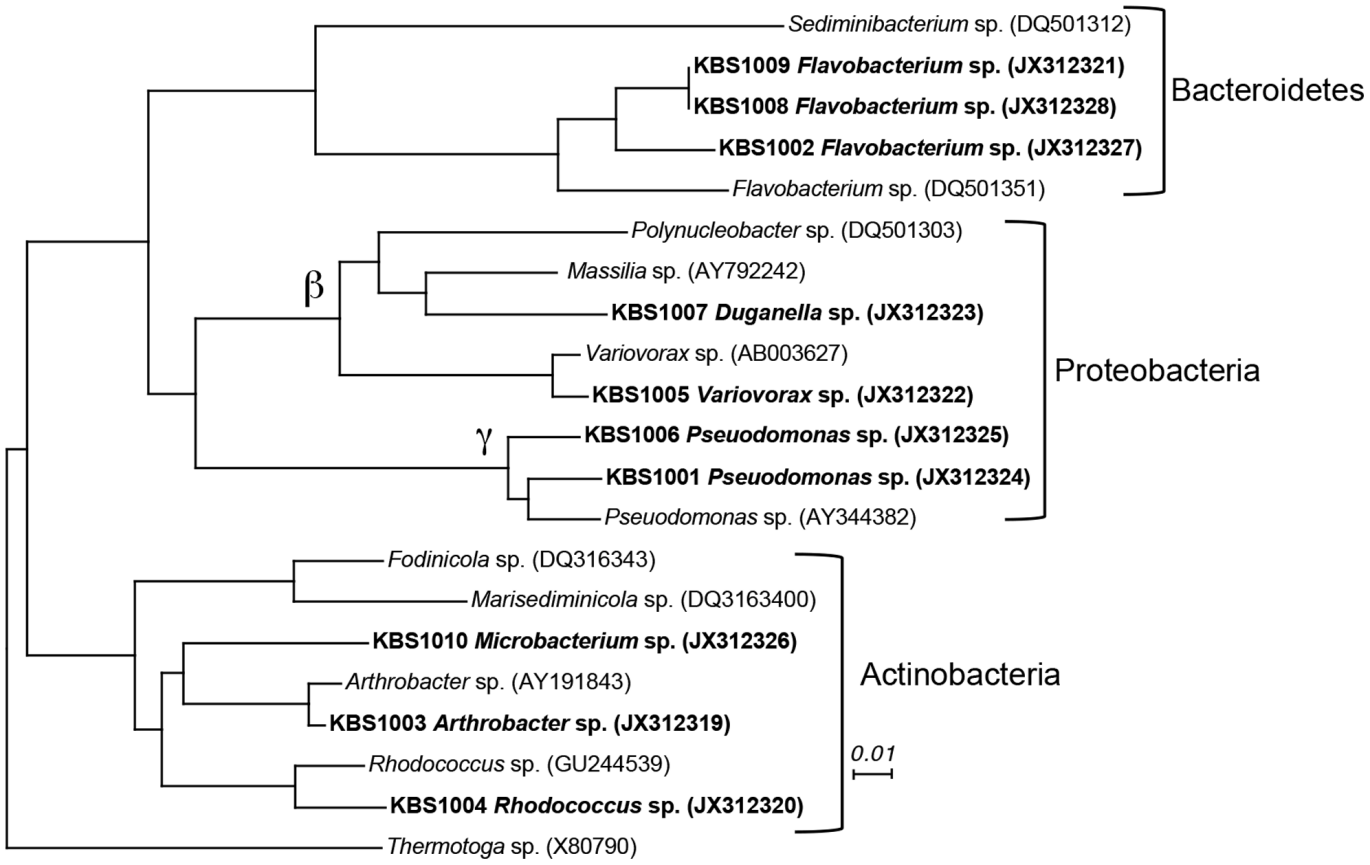
Heterotrophic bacteria grew on Super Hume. Phylogenetic relationship of bacteria cultivated on Super Hume as a sole carbon source (bold) along with reference sequences (non-bold). Trees were constructed from aligned 16S rRNA sequences using neighbor-joining methods. γ = γ-Proteobacteria and β = β-Proteobacteria. The scale bar represents the sequence dissimilarity. *Thermotoga* is an archaeon that was used as an outgroup.

We isolated and sequenced ten distinct bacterial isolates that were capable of growing on Super Hume as a sole carbon source. After our initial plating, colonies regrew when struck onto new agar plates containing Super Hume. However, we were not able to grow visibly turbid cultures in the liquid WC medium amended with Super Hume. The phylogenetic identities of the bacteria were not dissimilar to other bacteria that have been cultivated from freshwater ecosystems (Fig. 7). Three of the isolates belonged to the Bacterioidetes phylum, four belonged to the Proteobacteria pylum (two in the β-Proteobacteria class and two in the γ-Proteobacteria class), and three to the Actinobacteria phylum (Fig. 7).

### Biological Responses: Microbial Metabolism

**Figure 8:**
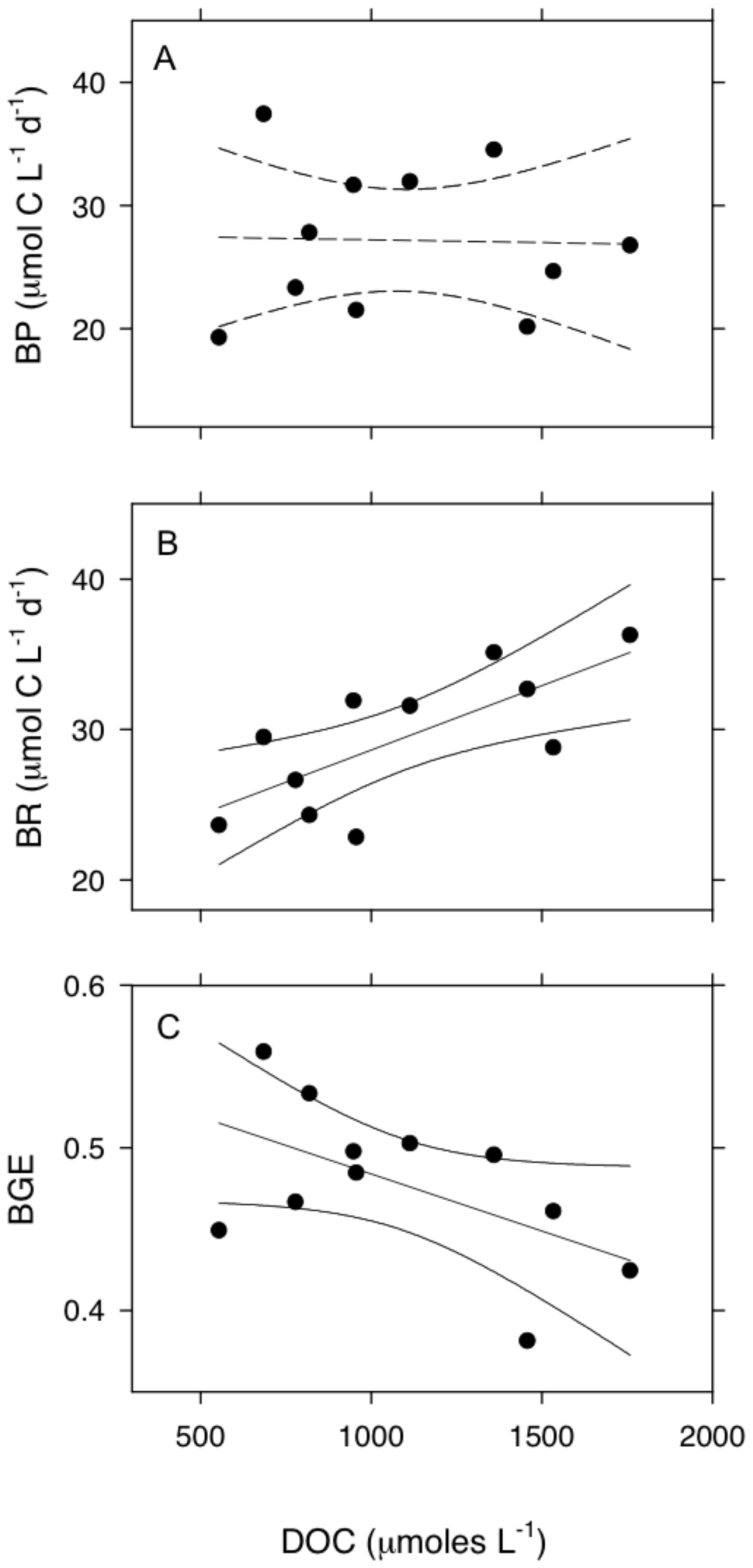
Microbial metabolism along a Super Hume gradient. We created a DOC gradient in a set of experimental ponds by altering the supply rate of Super Hume. There was no relationship between bacterial productivity (BP) and DOC (A), but bacterial respiration (BR) significantly increased (B) and bacterial growth efficiency (BGE) significantly decreased (C) along the DOC gradient. Regression lines represent predicted values and 95% confidence intervals for significant (solid lines) and non-significant (dashed lines) models.

Despite the growth of bacterial isolates on Super Hume agar plates, we did not observe a significant relationship between bacterial productivity (BP) and DOC concentration in the experimental ponds (r^2^ = 0.0008, F_1,10_ = 0.007, *P* = 0.94, Fig. 8a). In contrast, bacterial respiration (BR) significantly increased along the DOC gradient (r^2^ = 0.52, F_1,10_ = 9.85, *P* = 0.012, Fig. 8b).
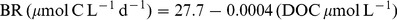
(5)


This change in BR resulted in a marginally significant decline in bacterial growth efficiency (BGE) along the DOC gradient (r^2^ = 0.30, F_1,10_ = 3.94, *P* = 0.079, Fig. 8c):
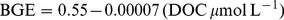
(6)


## Discussion

In this study, we evaluated a commercially available source of humic substances (Super Hume) for studying the effects of terrestrial DOC on the chemistry, physics, and biology of aquatic ecosystems. Similar to natural organic matter, Super Hume attenuated solar radiation in ways that are not only likely to affect the stress physiology of aquatic taxa, but also the metabolism of entire ecosystems. Super Hume may also provide insight into the effects of allochthonous resource subsidies on aquatic ecosystems. Specifically, our results suggest that Super Hume can enter aquatic food webs through 1) the uptake of dissolved organic constituents by microorganisms, and 2) the consumption of particulate fractions by larger organisms (e.g. crustacean zooplankton). Although the C:N and C:P ratios, potassium content, and flocculation rates could be high, Super Hume had properties that were very similar to natural sources of DOC found in terrestrial and aquatic ecosystems. Thus, Super Hume and other Leonardite-derived humic substances may be useful for addressing questions about the effects of terrestrial carbon export on aquatic ecosystems.

### Light Attenuation by Super Hume

The light-absorbing properties of Super Hume were comparable to those of natural DOC. Statistically, the parameters describing the relationship between Secchi Depth and DOC in a diverse set of north temperate lakes were similar to the parameters obtained from experimental ponds enriched with Super Hume (Fig. 2B). Likewise, when estimating light attenuation via color (a_440_), most observations from the Super Hume-enriched ponds fell within 95% prediction intervals associated with the color-DOC relationship observed for lakes. At high DOC concentrations, however, Super Hume absorbed more light than organic matter found in natural aquatic ecosystems (Fig. 2A). This difference could be due to unique chromophoric properties of Super Hume. Alternatively, the higher carbon-specific light absorbance could reflect features of the experimental ponds. For example, because our experiment took place over a relatively short period of time (4 months), Super Hume may have experienced less photobleaching than DOC found in natural lakes with longer residence times [62]. Our PARAFAC results provide additional insight into the spectral properties of Super Hume (Fig. 5), but more sophisticated optical analyses could help elucidate how DOC influences species interactions and ecosystem processes [63].

### Microbes Subsidized by Super Hume

Super Hume may provide insight into the mechanisms influencing the variation of allochthony among aquatic ecosystems [8]. Because most terrestrial carbon enters aquatic ecosystems in a dissolved form [64], it is often assumed – at least in lentic systems – that allochthony is influenced by the uptake of DOC by aquatic bacteria [65]. We isolated bacteria from a DOC-enriched pond that were capable of growing in the laboratory on Super Hume as the sole carbon source. These bacteria came from diverse phyla and were related to other taxa that have been recovered from lakes using cultivation-based approaches (see Fig. 7). Owing to the biases of culture-based methods, it is not surprising that our isolates were not closely related to lake bacteria that have been characterized using cultivation-independent molecular techniques [66]. Nevertheless, the isolates from this study provide unique opportunities to identify genomic and physiological traits that allow aquatic microbes to use recalcitrant carbon substrates. Last, our field data suggest that Super Hume stimulated bacterial respiration (Fig. 8), which increased linearly with increasing Super Hume loading rate, while bacterial productivity remained constant, which led to a slight decrease in bacterial growth efficiency along the gradient. Such findings are consistent with the view that anabolic and catabolic processes can be decoupled [67] and that terrestrial carbon may be preferentially allocated to respiration over growth.

### Is Super Hume Channeled Directly to Higher Trophic Levels?

Aquatic food webs can also be subsidized when non-microbial taxa consume terrestrial-derived particulate organic carbon (POC) [68]. Based on our size distribution analysis, 20% of Super Hume can be operationally defined as POC (>0.7 µm), which is comparable to the proportion of DOC and POC that enters natural lakes [69], [70]. This means that some of the Super Hume added to aquatic systems is potentially available for direct consumption by higher trophic levels, including filter-feeding zooplankton like *Daphnia* [71], [72]. We observed what appeared to be humic substances in the guts of gravid *D. pulex x pulicaria* from a Super Hume-enriched pond (Fig. 6a). This prompted us to conduct a life table experiment where we grew *Daphnia* on algae in treatments with or without Super Hume. Compared to the control, we documented that the age at first reproduction was reduced by one day and that there was a 10% increase in the intrinsic rate of increase for *Daphnia* in the Super Hume treatment (Fig. 6). This apparent stimulation of *Daphni*a growth by Super Hume could have been accompanied by changes in algal ingestion rates and assimilation rates, but there was no statistical support for this hypothesis. Additional experiments are needed to assess the interactions between terrestrial carbon inputs (DOC and POC) and autochthonous resources (e.g., algae) on the energetics and fitness of *Daphnia* and other consumer populations.

### Implications of Super Hume Flocculation

The fate of Super Hume may also be influenced by interactions between humic substances and other biogeochemical features of aquatic ecosystems. For example, we observed substantial variation in the flocculation rates of Super Hume among ecosystems; this variability is important for understanding the effects of terrestrial carbon loading on planktonic food webs and sediment carbon storage. In the experimental ponds, we estimated that approximately 5–12% of the total DOC pool was lost each day to flocculation. However, flocculation rates were almost an order of magnitude lower when measured in a set of lakes in northern Wisconsin. Physical forces and microbiological activity are known to affect flocculation rates [70], [73], but the aggregation of DOC into colloids and POC can be influenced by water chemistry, too. For example, alkaline water bodies tend to have higher concentrations of Ca^2+^ and Mg^2+^, which can promote DOC flocculation [74]. The background alkalinity of the experimental ponds was approximately 3 meq L^−1^, which is typical for inland water bodies of southwestern Michigan. In contrast, the lakes sampled in northern Wisconsin are ionically dilute and have much lower alkalinity (0.1 meq L^−1^) [75]. Therefore, ionic composition is one important biogeochemical characteristic that should be taken into consideration when attempting to target DOC concentrations via Super Hume additions in aquatic ecosystems.

### Opportunities for Isotopic Inference

Natural abundances of carbon isotopes are frequently used to gain insight into the trophic relationships and the degree of allochthony in aquatic ecosystems [76]. The δ^13^C of Super Hume is approximately −23‰, which is slightly enriched relative to the isotope signature of terrestrial carbon that typically enters north-temperate inland water bodies (−27‰) [17]. Owing to the overlap with other carbon endmembers, it is unlikely that that δ^13^C of Super Hume will be useful for resolving questions related to allochthony. However, carbon flow in aquatic ecosystems can also be inferred using ^14^C [77], [78]. The Δ^14^C of Super Hume is very depleted (−984.8‰), which means that it could be used in future studies to elucidate the effects of terrestrial carbon on aquatic food webs and ecosystem processes.

### Other Chemical Considerations

In general, the chemical constituents of Super Hume were similar to natural sources of dissolved organic matter (DOM) found in soils and aquatic ecosystems (Figs. 3, 4, 5). For example, py-GC/MS data suggest that Super Hume has a chemical signature that is similar to soil organic matter, which is enriched in aromatic, aliphatic, and nitrogen-bearing compounds, but also lignin, polysaccharides, and proteins. Super Hume did not group with DOC from a dystrophic lake (Brandywine) based on py-GC/MS, suggesting that the chemistry of terrestrial organic matter is modified by physical and biological processes after being delivered to aquatic ecosystems. We also analyzed Super Hume using a PARAFAC modeling approach [49], which allowed us to compare its fluorescence signal to 379 samples from aquatic ecosystems around the world. Almost all of the variation (99%) in our Super Hume sample could be explained using the PARAFAC model, which means that Super Hume did not have any anomalous spectrofluorimetric properties. Rather, the PARAFAC model revealed that Super Hume was enriched in quinones and other humic substances commonly found in terrestrial-derived organic carbon.

The nitrogen and phosphorus content of organic matter can be important for understanding the effects of resource quality on consumer populations and ecosystem processes [79]. Super Hume had high C:N (440) and C:P (10,128) ratios. In terrestrial systems, the C:N ratio and C:P ratios of bulk soil organic matter are typically much lower (14∶1 and 186∶1, respectively) [80]. In aquatic ecosystems, the C:N and C:P ratios of DOM can be slightly higher than what is reported for soils, especially when attempts are made to physically isolate autochthonous and allochthonous fractions of the DOM pool. For example, the C:N ratio of fulvic acids in the Suwanee River was ∼90∶1 [81], while the C:P ratio of fulvic acids in a Colorado stream were sometimes >3,500∶1 [82]. Based on these comparisons, Super Hume would be considered a low quality resource for aquatic consumers (e.g., microbes and zooplankton). However, this feature of Super Hume may provide opportunities for understanding the effects of DOM stoichiometry on aquatic ecosystems. For example, researchers could add different amounts or forms (e.g., inorganic and organic) of nitrogen and phosphorus to Super Hume-enriched systems to address questions related to DOM quality.

In general, experimental additions of Super Hume should contribute minimally to the concentration of cations and anions found in many aquatic and terrestrial ecosystems (Fig. 3). One exception is the relatively high potassium content of Super Hume, presumably due to the fact that commercial processing of humic substances from Leonardite involves an alkaline (i.e., KOH) extraction. Because it is major constituent of the cytosol and is involved in ion regulation, elevated potassium could affect aquatic biota in different ways. It is generally assumed that potassium limitation is rare [83], but there are instances where enrichment can stimulate the growth of aquatic taxa, including algae [84] and fungal pathogens [85]. In contrast, some studies have suggested the possibility of potassium toxicity. For example, potassium inhibited *Microcystis* (Cyanobacteria) populations at concentrations >2,800 µmol L^−1^ [86] and *Dinobryon* spp. (Chrysophyceae) at concentrations >383 µmol L^−1^ [87]. Other lines of evidence, however, suggest that phytoplankton are robust to a wide range of potassium concentrations. Chrysophyte biomass was not correlated to potassium concentrations in a survey of Swedish lakes [88]. In addition, we tested for evidence of inhibition by examining the relationship between algal biomass (chlorophyll *a*) and potassium in the US Environmental Protection Agency’s National Lake Assessment database (http://water.epa.gov/type/lakes/lakessurvey_index.cfm). After accounting for the correlation between total phosphorus (TP) and chlorophyll *a* (*r* = 0.75) in a large set of lakes (n = 1,070), there was no effect of potassium concentration (range = 1.4–36,114 µmol L^−1^) on algal biomass (*P* = 0.301, r^2^ = 0.001). In summary, the potassium associated with commercially available humic substances extracted with KOH could be important in certain systems, but was not problematic in the current study (e.g., Fig. 6).

### Caveats and Conclusions

In this study, we have explored the possibility of using a commercial source of humic substances to conduct browning experiments, and we have shown how this material is a good analog for natural DOC of terrestrial origin. Compared to natural DOC, Super Hume has similar organic chemical properties and optical effects on light transmission. Moreover, Super Hume was used by at least some heterotrophic bacteria and was not toxic to crustacean zooplankton (i.e., *Daphnia pulex x pulicaria*).

There are, however, a number of caveats that should be considered. First, the use of commercially available humic substances in ecotoxicology has been scrutinized because of certain distinct chemical properties [33], [35], which influence their interaction with DDT, PCBs, metals, and other contaminants [34]. Second, it is impossible for any single source of organic matter to capture the chemical and physical complexity of organic matter derived from diverse natural settings. Last, it is conceivable that the extraction and processing procedures may vary among companies or even among batches. For example, some companies add exogenous organic matter (e.g., seaweed) to their humic products; although these amendments may be beneficial for agricultural purposes, they risk confounding aquatic ecology experiments. As mentioned above, researchers should be aware of high potassium content, high C:N and C:P ratios, and possibly other attributes that were not considered in the current study. Nonetheless, commercially available humic substances like Super Hume may provide opportunities to address ecological questions using a fairly standardized product that can easily be obtained in large quantities at an affordable cost. Experimental additions of commercially available humic substances will allow researchers to gain insight into the complex physical, chemical, and biological responses that aquatic ecosystems might experience due to browning and other global change scenarios [16].
